# Sulphadoxine-pyrimethamine plus azithromycin may improve birth outcomes through impacts on inflammation and placental angiogenesis independent of malarial infection

**DOI:** 10.1038/s41598-019-38821-2

**Published:** 2019-02-19

**Authors:** Holger W. Unger, Annjaleen P. Hansa, Christelle Buffet, Wina Hasang, Andrew Teo, Louise Randall, Maria Ome-Kaius, Stephan Karl, Ayen A. Anuan, James G. Beeson, Ivo Mueller, Sarah J. Stock, Stephen J. Rogerson

**Affiliations:** 1 Department of Medicine at the Doherty Institute, University of Melbourne, Parkville, Victoria, Australia; 2 Central Clinical School and Department of Microbiology, Monash University, Victoria, Australia; 3 Papua New Guinea Institute of Medical Research, Goroka, Papua New Guinea; 4 Walter and Eliza Hall Institute, Parkville, Victoria, Australia; 5 Department of Medical Biology, University of Melbourne, Parkville, Victoria, Australia; 6 Australian Institute of Tropical Health and Medicine, James Cook University, Cairns, Australia; 7 Burnet Institute, Melbourne, Victoria, Australia; 8 Institut Pasteur, Paris, France; 9 Tommy’s Centre for Maternal and Fetal Health, MRC Centre for Reproductive Health, Queen’s Medical Research Institute, Edinburgh, UK

## Abstract

Intermittent preventive treatment with sulphadoxine-pyrimethamine (SP) and SP plus azithromycin (SPAZ) reduces low birthweight (<2,500 g) in women without malarial and reproductive tract infections. This study investigates the impact of SPAZ on associations between plasma biomarkers of inflammation and angiogenesis and adverse pregnancy outcomes in 2,012 Papua New Guinean women. Concentrations of C-reactive protein (CRP), α-1-acid glycoprotein (AGP), soluble endoglin (sEng), soluble fms-like tyrosine kinase-1 (sFlt-1) and placental growth factor (PlGF) were measured at enrolment and delivery in a trial comparing SPAZ to SP plus chloroquine (SPCQ). At antenatal enrolment higher CRP (adjusted odds ratio 1.52; 95% confidence interval [CI] 1.03–2.25), sEng (4.35; 1.77, 10.7) and sFlt1 (2.21; 1.09, 4.48) were associated with preterm birth, and higher sEng with low birthweight (1.39; 1.11,3.37), in SPCQ recipients only. Increased enrolment sFlt1:PlGF ratios associated with LBW in all women (1.46; 1.11, 1.90). At delivery, higher AGP levels were strongly associated with low birthweight, preterm birth and small-for-gestational age babies in the SPCQ arm only. Restricting analyses to women without malaria infection did not materially alter these relationships. Women receiving SPAZ had lower delivery AGP and CRP levels (p < 0.001). SPAZ may protect against adverse pregnancy outcomes by reducing inflammation and preventing its deleterious consequences, including dysregulation of placental angiogenesis, in women with and without malarial infection.

## Introduction

Adverse pregnancy outcomes including low birth weight (LBW, <2500 g), preterm birth (PTB, <37 gestational weeks) and small-for-gestational-age (SGA) are frequent in low- and middle-income countries (LMIC)^1^. Malaria, sexually transmitted infections, and urinary tract infections are common and contribute to adverse pregnancy outcomes in these settings^1–3^. The burden of LBW is highest in sub-Saharan Africa and South Asia^4^, regions where facilities to care for LBW babies are scarce and neonatal death commonly ensues^5^.

In pregnancy, inflammation, as measured through maternal plasma C-reactive protein (CRP) levels, has been associated with SGA^6^, pre-eclampsia^7^, and PTB^8^, with most evidence originating from high-income settings. In LMICs, important drivers of increased CRP include placental malaria, *Chlamydia trachomatis* infection, and chronic inflammation at distal sites (e.g. periodontitis)^9–11^. Spontaneous PTB is described as a multifactorial syndrome, and inflammation, whether sterile or as a result of infection, is thought to be an important contributing factor^12^.

Inflammation and angiogenesis are closely linked^13^. Malaria infection and concomitant inflammation have been associated with increased maternal serum levels of the antiangiogenic protein endoglin (sEng; expressed by vascular endothelium and syncytiotrophoblast) and reduced levels of proangiogenic placental growth factor (PlGF), resulting in placental vascular remodeling and fetal growth restriction^14^.

Intermittent preventive treatment in pregnancy (IPTp), the presumptive administration of antimalarials at antenatal clinic visits, decreases placental malaria and improves birthweights^15^. IPTp was introduced as many pregnant women with malaria are asymptomatic and point-of-care tests missed placental infections^16^. Currently recommended is monthly sulphadoxine-pyrimethamine (SP) from second trimester, but new IPTp candidate regimens are needed^17^. Two clinical trials of SP plus azithromycin (AZ) demonstrated reduction in the risk of PTB and LBW^18,19^. In Papua New Guinea (PNG), women randomised to three courses of SP and AZ (1 g twice daily for 2 days) had a lower risk of LBW (26% relative risk reduction, P = 0.005) and PTB (38%, P = 0.01) compared to control (single dose of SP plus chloroquine for 3 days at first antenatal visit, as per national policy)^18^. In Malawi, monthly courses of SP plus AZ (1 g) given at first and second treatment visits in addition to routine SP reduced the risk of LBW (relative risk reduction 39%, P = 0.04) and PTB (34%, P = 0.01) compared to women receiving two monthly SP treatments only^19^.

AZ is a macrolide with activity against *Plasmodium* spp. parasites as well syphilis, *C*. *trachomatis* and *Neisseria gonorrhoeae*, and urinary tract infections^20,21^. Structurally related to tacrolimus, it has immunomodulatory and anti-inflammatory properties, most studied in chronic lung diseases^22^. SP and SPAZ reduce LBW and PTB in the context of falling malaria prevalence, in areas with high-level resistance to SP, and in absence of malaria infection^23–25^. This suggests antibacterial and anti-inflammatory properties of SP and AZ may play a significant role.

Inflammation due to infectious and non-infectious processes may lead to adverse pregnancy outcomes and may do so it part by altering placental angiovasculogenesis. Surrogate measures of inflammation (CRP, α-1-Acid glycoprotein [AGP]) and angiogenesis sEng, soluble fms-like tyrosine kinase [sFlt-1], and PlGF are biomarkers that have been associated with pregnancy outcomes. We hypothesised that SPAZ prevents adverse pregnancy outcomes by reducing and preventing inflammation and dysregulation of placental angio- and vasculogenesis. We examined the relationship between biomarkers of inflammation and angiogenesis at antenatal enrolment or at delivery and adverse pregnancy outcomes. We then determined whether these relationships differed by treatment arm and persisted amongst women without malarial infection. This secondary analysis was performed drawing on data and samples of the aforementioned PNG-IPTp trial which was conducted at several health centres situated in Madang Province on the North Coast of PNG from 2009 to 2013^18^. The majority of pregnant women enrolled in the trial resided in rural and peri-urban areas where there is perennial transmission of *P*. *falciparum* and *P*. *vivax* malaria. The region is characterised by a high burden of LBW (17%), and a high prevalence of bacterial sexually transmitted infections and undernutrition in pregnancy^26–29^.

## Results

### Study population

**Figure 1:**
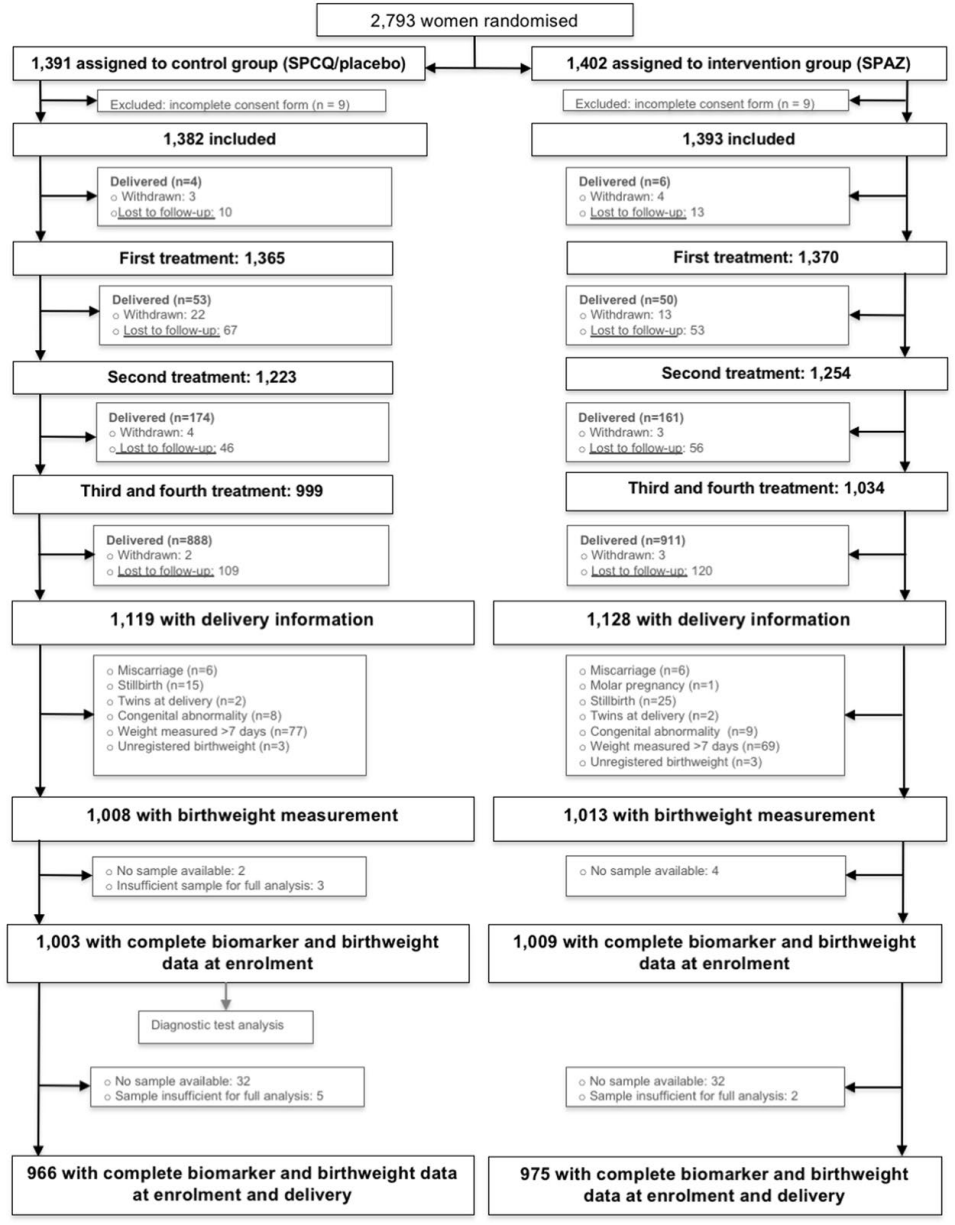
Participant flow chart. SPCQ, sulphadoxine-pyrimethamine plus chloroquine; SPAZ, sulphadoxine-pyrimethamine plus azithromycin.

Of 2,973 participants in the trial, 2,021 completed birthweight follow-up and delivered a live singleton congenitally normal infant^18^. Biomarker levels at enrolment were measured for 2,012 of these women (99.6%), and 1,941 (96.5%) had biomarker assessments at both enrolment and delivery (Fig. 1).

Women in each group had similar characteristics at enrolment (Table 1), apart from higher CRP levels amongst women randomised to SPAZ (p = 0.025).

### Pregnancy outcomes

The burden of adverse pregnancy outcomes was high. There were 303 women who delivered LBW infants (15.1%) (Table 1). Amongst 1,314 ultrasound-dated pregnancies, 8.7% (n = 114) had spontaneous PTB, and 24.5% (n = 322) were SGA. SPAZ reduced the risk of LBW and PTB. Women randomised to SPAZ had lower levels of CRP (P = 0.003) and AGP (P = 0.015) at delivery (Table 1).

**Table 1:** Cohort characteristics and birth outcomes, by treatment arm.

Variable	N	SPCQ	SPAZ	*P*
		(n = 1,003)	(n = 1,009)	
*Enrolment*				
Smoker	2,012	18.3	20.4	0.22
Malaria infection^a^	2,012	13.8	13.5	0.86
Primigravida	2,009	51.7	49.2	0.27
Haemoglobin (g/L)	1,927	97 (96, 98)	97 (96, 98)	0.41
Gestational age by ultrasound (weeks)	1,314	21.7 (21.4, 22.0)	21.8 (21.4, 22.1)	0.91
Mid-upper arm circumference (cm)	1,970	23.9 (23.8, 24.1)	24.0 (23.8, 24.2)	0.44
Maternal age (years)	2,012	24.7 (24.3, 25.0]	24.4 (24.0, 24.8]	0.38
CRP (mg/L)	2,012	1.3 (1.2, 1.4)	1.5 (1.4, 1.7)	0.025
AGP (mg/L)		222 (211, 232)	220 (209, 232)	0.85
sEng (pg/ml)		14.8 (14.1, 15.5)	14.6 (14.0, 15.4)	0.74
sFlt-1(ng/ml)		2.6 (2.5, 2.8)	2.5 (2.4, 2.7)	0.39
PIGF (pg/ml)		162 (155, 171)	156 (149, 164)	0.27
*Delivery*		(n = 966)	(n = 975)	
Low birthweight	2,012	17.5	12.7	0.003
Preterm birth (<37 weeks)	1,314	10.6	6.8	0.013
Small-for-gestational-age	1,314	25.1	23.9	0.61
Female baby	2,006	56.8	54.7	0.36
Birthweight (g)	2,012	2922 (510)	2964 (445)	0.05
Gestational age by ultrasound (weeks)	1,314	39.0	39.3	0.013
CRP (mg/L)	1,941	1.3 (1.2, 1.4)	1.0 (0.9, 1.1)	0.003
AGP (mg/L)		175 (167, 183)	162 (154, 169)	0.015
sEng (pg/ml)		23.0 (21.7, 24.3)	22.6 (21.4, 24.0)	0.75
sFlt-1 (ng/ml)		4.9 (4.5, 5.3)	4.8 (4.4, 5.2)	0.74
PlGF (pg/ml)		74.8 (71.6, 78.1)	76.3 (73.1, 79.6)	0.50

Continuous data are presented arithmetic mean (95% confidence interval) [clinical variables] or as geometric mean (95% confidence interval) [biomarker levels]. Categorical data are presented as %. *AGP*, α-1-acid glycoprotein; *CRP*, C-reactive protein; *PlGF*, placental growth factor; *sEng*; soluble endoglin; *sFlt-1*, soluble fms-like tyrosine kinase; *SPCQ*, sulphadoxine-pyrimethamine plus chloroquine; *SPAZ*, sulphadoxine-pyrimethamine plus azithromycin.

^a^Peripheral parasitaemia by light microscopy and/or real-time polymerase chain reaction (*Plasmodium falciparum*, *P*. *vivax*).

### Relationship between biomarker levels and clinical factors

At enrolment, women with clinical signs of infection (feeling unwell, history of self-reported fever in preceding 24 hours) and anaemia had higher CRP levels, and peripheral malaria infection was associated with increased CRP (P < 0.001), lower sFlt-1 (P = 0.017), and reduced levels of PlGF (P = 0.006) (Supplemental Tables 1, 2). Women with a mid-upper arm circumference < 23 cm (a clinical marker of undernutrition) had lower levels of CRP (P < 0.007) and higher levels of sFlt-1 (P = 0.018). A mean arterial pressure ≥ 90 mmHg at antenatal enrolment was associated with increased sEng (P < 0.001) (Supplemental Tables 1, 2).

At delivery, all malariometric indices were associated with increased levels of CRP, and active placental malaria with increased AGP (P = 0.012) (Supplemental Table 1). Evidence of past infection on placental histology was associated with lower levels of sEng (P = 0.003), sFlt-1 (P = 0.013) and reduced sFlt-1:PlGF ratios (P = 0.008) (Supplemental Table 2).

### Biomarker levels at enrolment and adverse pregnancy outcomes

Assessing all women, higher levels of sEng were associated with LBW and PTB, sFlt-1 with LBW, higher sFlt-1:PlGF ratios with LBW, and lower PlGF levels with SGA (Table 2). Stratification by trial arm revealed differences in associations between biomarker levels and adverse pregnancy outcomes. Significant associations were largely restricted to women who had been randomised to SPCQ; amongst them, higher enrolment CRP levels were associated with PTB, increases in sEng were associated with LBW and PTB, and higher levels of sFlt-1 with PTB (Table 2). Increasing sFlt-1:PlGF ratios were associated with LBW overall and by treatment arm.

**Table 2:** Adjusted odds ratios (aOR) for biomarker levels at enrolment (log-transformed) and adverse birth outcomes, overall and by trial arm.

Outcome	SPCQ	*P*	SPAZ	*P*	All	*P*
	aOR (95% CI)		aOR (95% CI)		aOR (95% CI)	
**Low birthweight** ^a^	**175/1**,**003**		**128/1009**		**303/2**,**012**	
CRP (mg/L)	1.14 (0.87, 1.48)	0.32	0.98 (0.72, 1.32)	0.87	1.05 (0.86, 1.27)	0.64
AGP (mg/L)	0.94 (0.58, 1.51)	0.81	1.70 (0.96, 3.01)	0.07	1.21 (0.84, 1.74)	0.30
sEng (pg/ml)	1.93 (1.11, 3.37)	0.022	1.23 (0.69, 2.20)	0.48	1.58 (1.07, 2.35)	0.024
sFlt-1 (ng/ml)	1.39 (0.89, 2.16)	0.15	1.70 (0.96, 2.99)	0.07	1.47 (1.04, 2.09)	0.028
PlGF (pg/ml)	0.70 (0.42, 1.16)	0.15	0.75 (0.42, 1.33)	0.32	0.75 (0.51, 1.09)	0.12
sFlt-1/PlGF	1.48 (1.04, 2.20)	0.030	1.54 (1.02, 2.33)	0.040	1.46 (1.11, 1.90)	0.006
**Preterm birth** ^b^	**69/649**		**45/665**		**114/1**,**314**	
CRP (mg/L)	1.52 (1.03, 2.25)	0.035	0.86 (0.53, 1.39)	0.54	1.20 (0.86, 1.62)	0.24
AGP (mg/L)	0.92 (0.46, 1.86)	0.80	0.99 (0.43, 2.24)	0.97	0.96 (0.57, 1.62)	0.86
sEng (pg/ml)	4.35 (1.77, 10.7)	0.001	1.37 (0.52, 3.62)	0.53	2.49 (1.31, 4.74)	0.006
sFlt-1 (ng/ml)	2.21 (1.09, 4.48)	0.028	1.00 (0.41, 2.41)	0.99	1.64 (0.96, 2.82)	0.07
PlGF (pg/ml)	1.37 (0.59, 3.17)	0.47	0.66 (0.23, 1.87)	0.43	1.02 (0.53, 1.95)	0.95
sFlt-1/PlGF	1.44 (0.83, 2.51)	0.20	1.19 (0.61, 2.33)	0.61	1.34 (0.88, 2.05)	0.17
**SGA** ^b^	**163/649**		**159/665**		**322/1**,**314**	
CRP (mg/L)	1.05 (0.79, 1.39)	0.74	0.94 (0.70, 1.25)	0.68	0.98 (0.80, 1.19)	0.84
AGP (mg/L)	1.04 (0.64, 1.70)	0.87	1.36 (0.82, 2.24)	0.24	1.18 (0.84, 1.67)	0.35
sEng (pg/ml)	1.07 (0.61, 1.89)	0.85	1.03 (0.58, 1.81)	0.94	1.07 (0.78, 1.59)	0.78
sFlt-1 (ng/ml)	0.97 (0.59, 1.60)	0.90	1.12 (0.66, 1.88)	0.62	1.04 (0.72, 1.49)	0.83
PlGF (pg/ml)	0.54 (0.29, 1.01)	0.05	0.61 (0.33, 1.13)	0.12	0.59 (0.38, 0.91)	0.016
sFlt-1/PlGF	1.27 (0.85, 1.90)	0.25	1.34 (0.90, 2.00)	0.16	1.29 (0.97, 1.71)	0.08

*AGP*, α-1-acid glycoprotein; *CI*, confidence interval; *CRP*, C-reactive protein; *PlGF*, placental growth factor; *sEng*; soluble endoglin; *sFlt-1*, soluble fms-like tyrosine kinase; *SPCQ*, sulphadoxine-pyrimethamine plus chloroquine; *SPAZ*, sulphadoxine-pyrimethamine plus azithromycin. *NS*, not significant, defined as p ≥ 0.05.

^a^Odds ratios and 95% confidence intervals were estimated using logistic regression and adjusted for sex, bed net use, mid-upper arm circumference, recruitment clinic, height, partner’s work status, number of antenatal visits, timing of birthweight measurement, and fundal height at biomarker measurement.

^b^Odds ratios and 95% confidence intervals were estimated using logistic regression and adjusted for sex, bed net use, mid-upper arm circumference, recruitment clinic, height, partner’s work status, number of antenatal visits, and gestational age at biomarker measurement by ultrasound.

When we examined associations between abnormal biomarker levels at enrolment and birthweight (continuous variable) (Supplemental Table 3), women with elevated CRP levels (≥5.0 mg/L) who were randomised to SPCQ had an adjusted mean reduction in birthweight of 96 g (95% CI 13, 179) (P = 0.023). No other significant associations were observed.

### Relationship of biomarker levels at birth with treatment arm and adverse pregnancy outcomes

At birth biomarker data were available for 966 women (627 with PTB data) in the SPCQ arm, and 975 (646 with PTB data) in the SPAZ arm.

Women randomised to SPAZ had lower CRP and AGP levels at delivery (Table 1).

**Figure 2:**
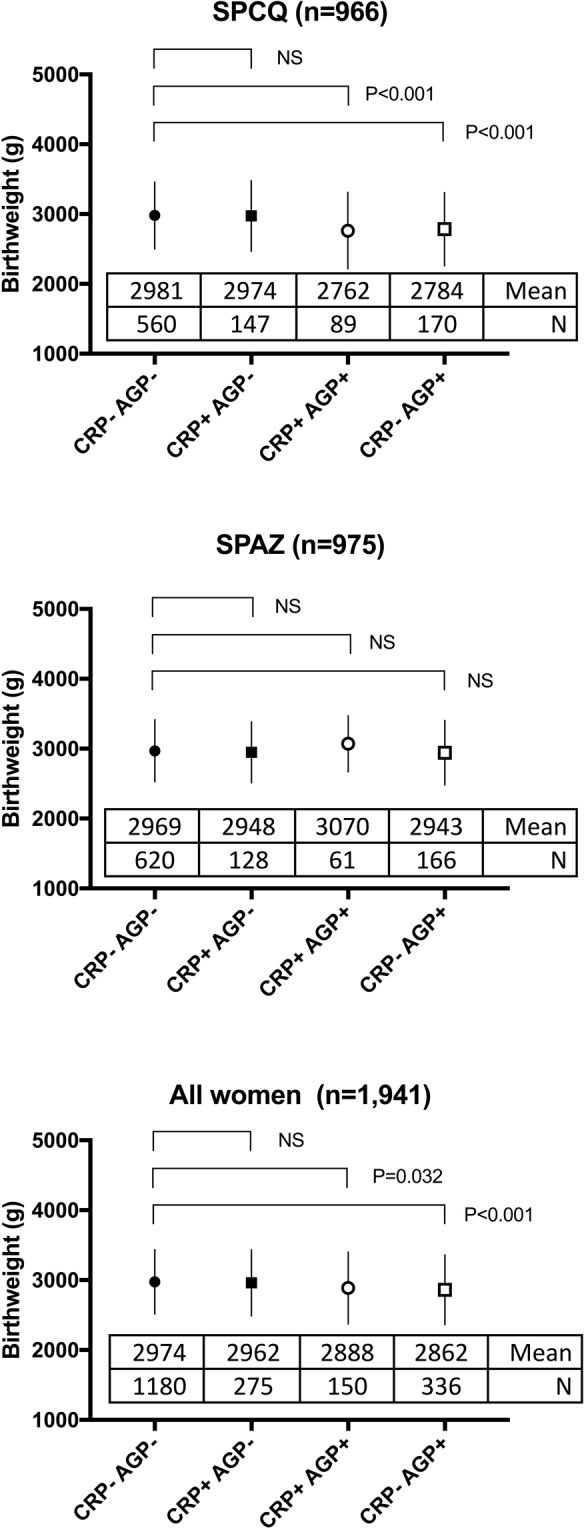
Association between inflammation category at delivery and birthweight, by treatment arm and overall. Figures indicate mean (circles or squares) and standard deviation (whiskers). Numbers show mean birthweight (g) and numbers of women in each group, based on presence or absence of elevation in C-reactive protein (CRP) or α-1-acid glycoprotein (AGP). SPAZ; sulphadoxine-pyrimethamine plus azithromycin; SPCQ, sulphadoxine-pyrimethamine plus chloroquine.

Higher levels of AGP at delivery were strongly associated with LBW, PTB and SGA. Upon stratification by trial arm, these associations were confined to women randomised to SPCQ (Table 3). Most pronounced effects on mean birthweight were observed amongst women with an elevated AGP at birth: birthweight reductions in women with raised AGP levels were similar in women with normal and concomitantly raised CRP levels (Fig. 2). When stratifying by treatment arm raised AGP levels were associated with pronounced reductions in birthweight (adjusted mean difference 180 g, 95% CI 111, 251) amongst SPCQ recipients (Fig. 2). A raised CRP was associated with reduced risk of PTB amongst SPAZ recipients (0.52; 0.28, 0.95; P = 0.035).

**Table 3:** Adjusted odds ratios (aOR) for biomarker levels at delivery (log-transformed) and adverse birth outcomes, overall and by trial arm.

Outcome	SPCQ	*P*	SPAZ	*P*	All	*P*
	aOR (95% CI)		aOR (95% CI)		aOR (95% CI)	
**Low birthweight** ^a^	**168/966**		**124/975**		**292/1**,**941**	
CRP (mg/L)	1.17 (0.91, 1.51)	0.23	0.97 (0.73, 1.30)	0.85	1.10 (0.91, 1.33)	0.32
AGP (mg/L)	2.40 (1.38, 4.18)	0.002	1.21 (0.67, 2.22)	0.53	1.79 (1.20, 2.68)	0.004
sEng (pg/ml)	1.79 (1.12, 2.88)	0.016	1.61 (0.95, 2.72)	0.08	1.69 (1.19, 2.40)	0.003
sFlt-1 (ng/ml)	1.05 (0.76, 1.44)	0.73	1.42 (0.95, 2.11)	0.08	1.18 (0.92, 1.51)	0.18
PlGF (pg/ml)	0.65 (0.36, 1.17)	0.14	0.51 (0.25, 1.03)	0.06	0.58 (0.37, 0.91)	0.018
sFlt-1/PlGF	1.18 (0.87, 1.60)	0.27	1.62 (1.12, 2.34)	0.010	1.34 (1.06, 1.69)	0.014
**Preterm birth** ^b^	**68/627**		**44/646**		**112/1**,**273**	
CRP (mg/L)	1.27 (0.85, 1.91)	0.22	0.69 (0.42, 1.13)	0.14	1.02 (0.76, 1.39)	0.87
AGP (mg/L)	5.27 (2.17, 12.08)	<0.001	0.73 (0.29, 1.82)	0.48	2.10 (1.14, 3.88)	0.017
sEng (pg/ml)	2.12 (1.05, 4.26)	0.036	2.59 (1.04, 6.46)	0.041	2.22 (1.28, 3.82)	0.004
sFlt-1 (ng/ml)	1.11 (0.68, 1.82)	0.67	2.10 (1.08, 4.10)	0.029	1.41 (0.96, 2.07)	0.08
PlGF (pg/ml)	0.34 (0.12, 0.95)	0.0393	0.46 (0.14, 1.50)	0.20	0.37 (0.17, 0.80)	0.011
sFlt-1/PlGF	1.42 (0.89, 2.28)	0.14	2.27 (1.23,4.17)	0.008	1.71 (1.19, 2.46)	0.004
**SGA** ^**b**^	**160/627**		**155/646**		**315/1**,**273**	
CRP (mg/L)	1.15 (0.88, 1.52)	0.30	1.01 (0.76, 1.35)	0.87	1.07 (0.88, 1.31)	0.46
AGP (mg/L)	2.93 (1.60, 5.36)	<0.001	1.23 (0.70, 2.14)	0.47	1.84 (1.23, 2.75)	0.003
sEng (pg/ml)	1.57 (0.98, 2.50)	0.05	1.11 (0.70, 1.76)	0.72	1.32 (0.95, 1.83)	0.10
sFlt-1 (ng/ml)	1.12 (0.79, 1.58)	0.51	1.16 (0.81, 1.67)	0.45	1.13 (0.88, 1.44)	0.33
PlGF (pg/ml)	0.90 (0.48, 1.67)	0.73	0.91 (0.48, 1.73)	0.77	0.91 (0.59, 1.42)	0.68
sFlt-1/PlGF	1.14 (0.82, 1.57)	0.42	1.16 (0.83, 1.62)	0.40	1.14 (0.91, 1.43)	0.27

*AGP*, α-1-Acid glycoprotein; *CI*, confidence interval; *CRP*, C-reactive protein; *PlGF*, placental growth factor; *sEng*; soluble endoglin; *sFlt-1*, soluble fms-like tyrosine kinase; *SPCQ*, sulphadoxine-pyrimethamine plus chloroquine; *SPAZ*, sulphadoxine-pyrimethamine plus azithromycin. *NS*, not significant, defined as p ≥ 0.05.

^a^Odds ratios and 95% confidence intervals were estimated using logistic regression and adjusted for sex, bed net use, mid-upper arm circumference, recruitment clinic, height, partner’s work status, number of antenatal visits, and timing of birthweight measurement.

^b^Odds ratios and 95% confidence intervals were estimated using logistic regression and adjusted for sex, bed net use, mid-upper arm circumference, recruitment clinic, height, partner’s work status, and number of antenatal visits.

Angiogenic markers exhibited differential associations with adverse birth outcomes when assessed by trial arm. Higher levels of sEng associated with LBW and PTB overall, with LBW amongst SPCQ recipients, and with PTB in both SPCQ and SPAZ recipients. Higher sFlt-1:PlGF ratios were associated with LBW and PTB amongst SPAZ recipients only (Table 3).

**Figure 3:**
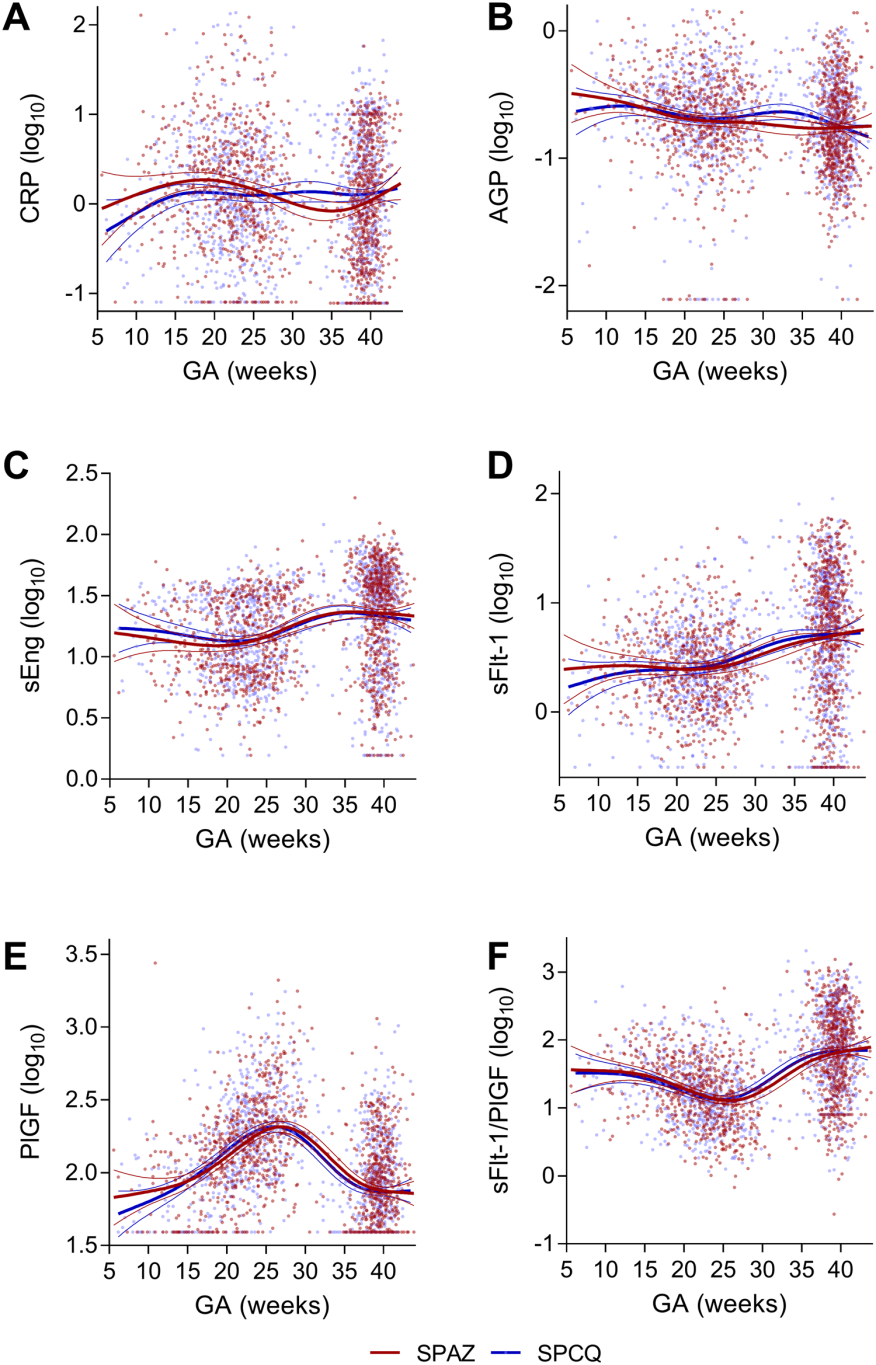
Longitudinal changes of biomarker levels according to treatment arm. Biomarkers levels (log_10_) measured at enrolment and delivery (1,273 pairs) were plotted by gestational age at measurement. (**A**) c-reactive protein (CRP); (**B**) α-1-acid glycoprotein (AGP); (**C**) soluble endoglin (sEng); (**D**) soluble fms-like tyrosine kinase (sFlt-1); (**E**), placental growth factor (PlGF); and (**F**) sFlt-1/PlGF. Curves indicate the best fit and 95% confidence bands of the general additive models. Data points and regression lines are coloured by treatment arm (red, sulphadoxine-pyrimethamine plus azithromycin, SPAZ; blue, sulphadoxine-pyrimethamine plus chloroquine, SPCQ).

Multilevel linear mixed effects models did not show any significant differences in biomarker levels with gestational age between treatment arms. Statistically significant differences between treatment arms for AGP (and CRP, data not shown) were noted in the 31–35 gestational weeks range (Supplemental Fig. 1). Biomarker levels were further described pictorially using generalised additive models (Fig. 3).

### Associations between biomarker levels and adverse pregnancy outcomes in women without malarial infection

Malaria infection may alter biomarker levels, and differences in antimalarial treatment efficacy may explain trends observed in Tables 2 and 3. In order to investigate the hypothesis that SPAZ reduces LBW through mechanisms other than malaria prevention and treatment we reassessed associations between biomarker levels at enrolment or delivery and adverse pregnancy outcomes amongst women who tested negative for malaria infection. Malaria infection was defined as *P*. *falciparum* and/or *P*. *vivax* detected at least once during pregnancy. Enrolment biomarker data from 1,540 women and delivery biomarkers from 1,481 women were analysed (Supplemental Tables 4,5).

Most associations observed in the overall cohort persisted in this subset of women unlikely to have had malaria infection (Supplemental Table 4). At enrolment and amongst SPCQ recipients, higher levels of sEng (adjusted odds ratio [aOR] 3.77; 95% CI 1.32, 10.7; P = 0.030; n = 484), CRP (2.30; 1.39, 3.79; P = 0.001), and sFlt-1 (2.50; 1.10, 5.60; P = 0.029) were associated with PTB. Higher enrolment sFlt-1:PlGF ratio was associated with LBW overall (1.54; 1.13, 2.10; P = 0.006), and amongst SPCQ recipients (1.56; 1.02, 2.37; P = 0.039). Higher levels of PlGF were associated with reduced odds of SGA in all women (0.56; 0.34, 0.94; P = 0.026).

Similarly, at delivery higher levels of AGP were associated with LBW (aOR 2.47, 95% CI 1.29, 4.73), P = 0.007), PTB (4.27; 1.53, 11.7; P = 0.005) and SGA (3.94; 1.91, 8.14; P <0.001) amongst SPCQ recipients without malaria infection (Supplemental Table 5). Higher levels of sEng were associated with LBW (2.15; 1.40, 3.29; P = 0.001) and PTB (3.06; 1.55, 6.07; P = 0.001) in all women, and with SGA amongst SPCQ recipients (1.82; 1.04, 3.18; P = 0.035). Higher levels of PlGF associated with reduced odds of LBW in all women (0.53; 0.31, 0.91; P = 0.021), and with reduced odds of PTB amongst SPCQ recipients (0.29; 0.09, 0.97; P = 0.045), and higher sFlt-1:PlGF ratios were associated with LBW amongst SPAZ recipients (1.70; 1.10, 2.63; P = 0.017).

### Prediction of adverse pregnancy outcomes

The role of biomarker measurements at first antenatal visit as tests to predict LBW, PTB or SGA was assessed amongst 1,003 women randomised to SPCQ (Supplemental Table 6). AUC readings for individual biomarkers at enrolment in relation to LBW, PTB or SGA were all below < 0.65, indicating poor diagnostic performance. The highest AUC, for the prediction of PTB, was observed for sEng (0.631). Combinations of biomarkers did not markedly improve the AUC, and neither did the addition of measured clinical factors, such as mid-upper arm circumference (all AUC <0.7).

## Discussion

Biomarkers of inflammation, infection and angiogenesis at first antenatal visit and delivery were associated with adverse pregnancy outcomes in women enrolled in a randomised controlled trial of malaria prevention in PNG. Most associations of biomarker levels at enrolment with adverse pregnancy outcomes were observed amongst women who were randomised to SPCQ, the control treatment. For instance, elevated levels of CRP, sEng, and sFlt-1 at antenatal enrolment were associated with PTB only amongst SPCQ recipients. At delivery, SPAZ was associated with reduced maternal serum levels of CRP and AGP. Raised delivery levels of AGP were strongly associated with LBW, PTB and a marked reduction in mean birthweight in SPCQ recipients only.

SPAZ demonstrated superior antimalarial efficacy compared to SPCQ^18^, and differences in associations between biomarkers and adverse pregnancy outcomes may be attributable to differential antimalarial efficacy alone. However, most treatment-specific associations between biomarkers and birth outcomes persisted in analyses that excluded women with detectable malaria infection. This suggests that SPAZ ablates other, malaria-independent, pathological inflammatory and angiogenic processes. These may include clearance of other causes of inflammation, such as sexually transmitted, reproductive tract and urinary tract infections^20^; preventing or treating ascending infection or infections at distal sites, such as periodontal disease^30^; or impacts on the vaginal or gut microbiome^31^. Additionally, azithromycin has anti-inflammatory actions that may decrease placental inflammation of infectious origin or indeed treat sterile inflammatory conditions^32^, thereby improving fetal growth and prolonging pregnancy duration.

Women in the control arm received once-off SPCQ treatments, whereas women in the SPAZ arm also received second and third treatments. Higher numbers of SP doses are associated with improved birth outcomes after adjusting for antimalarial efficacy, and SP is known to have antibacterial properties that may be of benefit^23^. The trial design precluded assessment of the possible independent impacts of AZ and SP on biomarker levels and adverse pregnancy outcomes. A trial of SPAZ in Malawi, which compared it to SP-IPTp rather than SPCQ, confirmed benefit of adding AZ to SP-IPTp for prevention of LBW and PTB^19^. A new trial which compares IPTp with dihydroartemisinin-piperaquine with or without AZ against SP-IPTp will permit further assessment of AZ-specific benefits for pregnancy outcomes and impacts on biomarkers (Clinicaltrials.gov NCT03208179).

A meta-analysis of the role of presumptive antibiotic treatment in second and third trimester for the prevention of PTB suggested no benefit^33^. However, trials such as the PNG trial were excluded as women in the control arm received active treatment^19^. The only LMIC trial of AZ for the prevention of PTB included in the meta-analysis showed no benefit^34^, but arguably the study should also have been excluded as women in the control arm received SP-IPTp as part of routine antenatal care. It is likely that the contribution of infection to PTB is high in low-income settings, and that the positive effect of SPAZ for PTB prevention in our trial is mediated through a broader reduction in the infective or inflammatory burden in participants. A reappraisal of evidence from high PTB burden settings on the relationship between use of agents with antibacterial and anti-inflammatory activity (including IPTp) and pregnancy outcomes appears warranted.

Presence of inflammation at either enrolment or delivery, indicated by levels of CRP and AGP, was associated with adverse outcomes amongst women who did not receive SPAZ. At enrolment, CRP was more strongly associated with PTB, whilst at delivery raised levels of AGP most strongly associated with LBW and PTB. Chronic inflammatory processes, which AGP is thought to reflect more prominently given its slower rise and longer half-life^35^, may be the main driver for events such as PTB; one possible process is chorioamnionitis. Biomarker levels at enrolment, alone or in combination, demonstrated only poor to moderate diagnostic performance.

Low PlGF levels and high sFlt-1:PlGF ratios in third trimester, which are used to predict pre-eclampsia, may be useful to identify women at risk of other adverse pregnancy outcomes^36,37^. This study finds that lower levels of PlGF at enrolment were associated with SGA, and at delivery, lower PlGF levels and increased sFlt-1:PlGF ratios, together with increased sEng, were associated with LBW and PTB. When stratified by treatment arm, sEng and sFlt-1 elevations at enrolment were associated with PTB only in SPCQ recipients, suggesting that SPAZ abolishes or ablates some pathological angiogenic processes. By contrast, increased sFlt-1:PlGF ratios and increased sFlt-1 were only significantly associated with PTB in SPAZ recipients, possibly reflecting residual disturbances affecting placental function that are independent of infection and inflammation (and thus cannot be prevented/abolished by SPAZ).

The strengths of this study include its setting (low-income, high burden), large sample size, and its part-prospective design and adjustment for confounding factors. To our knowledge this is the first study to assess the impact of antimalarial interventions on biomarker associations with adverse pregnancy outcomes. It allows further insight into the mechanisms of action of SPAZ, contributes to the growing body of evidence of secondary effects of IPTp regimens^29^, and provides further evidence for the role of inflammation and disturbed placental angiovasculogenesis in causing adverse pregnancy outcomes. Results of this research may be most applicable to LMICs, in particular the role of prophylactic antibiotic therapy, yet further evaluation of anti-inflammatory drugs may also be warranted in high-income settings.

Cross-sectional analysis of associations between biomarker levels at delivery and birth outcomes precludes causal inference. Associations at delivery may be biased as levels of sEng, sFlt-1 and PlGF are known to change in a non-linear fashion with advancing gestational age^38^. However, CRP levels may be comparatively stable during pregnancy^39,40^, and the mean difference in gestational age between treatment arms was less than two days. Biomarker levels were only measured twice during pregnancy, limiting longitudinal analysis of changes in biomarker levels during gestation. Statistically significant differences in levels between treatment arms were observed for both AGP and CRP at the 31–35 gestational weeks range (Fig. 3, Supplemental Fig. 1). However, bias is possible as more measurements were obtained from SPCQ recipients during this gestational age range given they were more likely to delivery preterm.

The study has other limitations. First, due to the imprecision associated with estimating gestational age using clinical measures such as last menstrual period in our setting^41^, PTB and SGA analyses were restricted to ultrasound-dated pregnancies (65% of women), potentially introducing selection bias. Some pregnancies were dated in second trimester, which could have overestimated the incidence of PTB. Second, the low prevalence of HIV infection in our clinics (~1%) precluded assessment of its contribution to adverse outcomes^18^. Furthermore, infections other than malaria (e.g. sexually transmitted infections, chorioamnionitis) were not assessed in detail to contribute to this analysis. In addition, women randomised to SPAZ had higher CRP levels at antenatal enrolment compared to SPCQ recipients, which may have exaggerated the impact of SPAZ on birth outcomes. Lastly, given the exploratory nature of the research we opted to present p-values that are unadjusted for multiple comparisons. This requires careful interpretation of the data in view of the increased potential for type 1 error.

Limited progress has been made with regards to the prevention of LBW and spontaneous PTB, in particular in LMICs. SPAZ significantly reduces the risk of both LBW and PTB and may do so in part through reducing inflammation and improving placental angiovasculogenesis and function. While the mechanisms by which these agents improve outcomes need definition, further exploration of their therapeutic potential in LMICs is required. The ability of prophylactic antimicrobial therapy in second and third trimester of pregnancy to prevent adverse birth outcomes may depend on the type of antibiotic, dosing regimens, anti-inflammatory properties, and maternal burden of infection and inflammation. Evidence from malaria prevention trials must not be ignored, and further high-quality research from LMICs is required, given the impact such interventions could have in preventing PTB and LBW in these high-burden settings. This includes measuring the impact of IPTp with SPAZ or dihydroartemisinin-piperaquine with AZ on levels of markers of inflammation and angiovasculogenesis assessed longitudinally during pregnancy until delivery.

## Materials and Methods

### Study design

We analysed samples and data collected as part of a randomised controlled trial of IPTp with SPAZ in PNG (Clinicaltrials.gov, NCT01136850)^18^. We assessed the relationship between inflammatory/angiogenic marker levels at antenatal enrolment or delivery with birth outcomes in all women and women without malaria, and by treatment arm.

### Study site and participants

The study took place from November 2009 until February 2013 at health centres in Madang Province, a malaria-endemic area with a high burden of adverse pregnancy outcomes on the north coast of PNG^18^. Healthy pregnant women below 26 weeks’ gestation by symphysis fundal height were randomized to either three courses of SP (3 tablets [500/25 mg] given once) and AZ (2 tablets [500 mg] twice daily for 2 days), given at minimum intervals of 4 weeks; or a clearance course of SP and chloroquine (3 or 4 tablets [150 mg], daily for 3 days) at enrolment, followed by monthly courses of placebo equivalent. Women received insecticide-treated bed nets and iron-folate supplementation. Biomarker studies were undertaken amongst women who completed follow-up for birthweight and who gave birth to a congenitally normal singleton infant ≥22 gestational weeks (or ≥500 g if not ultrasound-dated)^42^. Gestational age at delivery was estimated by ultrasound using the earliest scan available to date the pregnancy^18^. Analyses evaluating the outcome measures PTB and SGA were restricted to women with ultrasound-dated pregnancies (1,314 of 2,012 women [65.3%]), given clinical estimates of gestational age are prone to significant imprecision in our setting^41^.

### Laboratory analyses

We measured the acute-phase proteins C-reactive protein (CRP) and α-1-Acid glycoprotein (AGP), and angiogenic factors soluble fms-like tyrosine kinase (sFlt-1), PlGF and sEng on stored plasma (thawed from −80 °C) of maternal venous blood samples from enrolment and delivery^14^. We additionally calculated sFlt-1:PlGF ratios^43^.

We used commercially available enzyme-linked immunosorbent assays (ELISA) (Human Quantikine ELISA kits; R&D Systems, Minneapolis, MN, USA), according to manufacturer’s instructions. Human plasma samples were diluted with BSA-PBS (sEng: 1:25, PIGF and sFlt1: 1:5; CRP: 1:10,000; AGP: 1:1,000,000) and assayed in duplicates. Optical densities were read at 450 nm and 540 nm on a Fluostar Omega microplate reader (BMG Labtech, Ortenberg, Germany). Sample concentrations were interpolated from a standard curve and multiplied by the dilution factor. Samples above or below the standard curve, or with duplicates varying by >20%, were repeated, with dilution adjustments if required.

### Statistical analysis

Univariate comparisons of categorical data were performed using the Chi-squared test or Fisher’s exact test, as appropriate. Univariate comparisons of continuous parametric data (including normalised data) were performed using the Student’s t-test, and non-parametric data using the Mann Whitney-U test or Wilcoxon signed-rank test, as appropriate.

Logistic regression analyses were performed to evaluate associations between biomarker levels at enrolment or delivery and adverse birth outcomes (PTB, SGA, LBW). Biomarker distribution exhibited a strong positive skew. Biomarker data were log(10) transformed to normalise the data prior to inclusion in the model as a continuous predictor^44^. Biomarker levels were presented as geometric means (95% confidence intervals). P < 0.05 was considered significant.

SGA was defined as a birthweight below the 10^th^ centile of the Intergrowth-21^45^. Models were adjusted *a priori* for factors previously found to be associated with LBW in the cohort (bed net use, mid-upper arm circumference, recruitment clinic, maternal height, socio-economic status, number of antenatal visits, sex of the baby, timing of birthweight measurement)^18^. We additionally adjusted for gestational age at enrolment to account for fluctuations in biomarker levels during pregnancy^38,46^. Associations between biomarkers and outcomes were assessed overall, and by trial treatment arm. When using birthweight as the outcome measure, biomarker levels were included in the linear regression model as dichotomised variables: a raised CRP was defined as a CRP ≥ 5 mg/L^47^, elevated levels of AGP, sFlt-1 and sEng were defined as readings in the 4th quartile, and a low PlGF as the first quartile. A sFlt-1:PlGF ratio ≥ 38 was defined as elevated^43^. We additionally evaluated combinations of CRP and AGP levels, given their differing temporal dynamics^48^.

General additive models were fitted to the longitudinal data of biomarkers measured at enrolment and birth and gestational age, separately for SPAZ and SPCQ treatment arms. Curve fitting was conducted using R and its ‘gam’ package. Generalised additive models were allowed 4 degrees of freedom. Multilevel linear mixed effects models (Stata 13) were used to assess changes in biomarker levels with gestational age and to evaluate whether there were any differences in the biomarker level-gestational age relationship between treatment arms.

Because SPAZ significantly reduced peripheral and placental blood parasitemia and active malaria infection on placental histology^18^, and active placental infection was associated with LBW and PTB in the cohort^49^, we investigated whether associations between biomarkers and adverse outcomes according to treatment arm persisted in women unlikely to have had malaria. Specifically, we excluded all women who at least once tested positive during pregnancy for *P*. *falciparum* or *P*. *vivax* on peripheral or placental blood smear, and/or polymerase chain reaction of peripheral and placental blood, or when available, placental histology (active infection)^16,49^. Peripheral blood testing was performed at enrolment, at subsequent IPTp visits, and at delivery^18^.

Diagnostic characteristics of biomarkers at enrolment to predict LBW, PTB, and SGA were assessed amongst women subsequently randomised to SPCQ and included receiver-operator curve characteristics (area under the curve [AUC]), the Youden index, sensitivity, specificity, positive and negative predictive values and positive and negative likelihood ratios.

### Ethical considerations

Ethical approval for this study was obtained from the PNG Institute of Medical Research (PNGIMR) Institutional Review Board, the PNG Medical Research Advisory Council, and the Melbourne Health Human Research Ethics Committee (Australia). The study was conducted in accordance with Good Clinical Practice guidelines (ICH GCP E6). All participants provided written informed consent.

## Supplementary information

Supplemental Information

## Data Availability

Parent trial data are available from the WWARN data repository (http://www.wwarn.org/working-together/sharing-data/accessing-data) for researchers who meet the criteria for access to confidential data. Biomarker data are available from the corresponding author by written request.
