# Supplementary material for: Sulphadoxine-pyrimethamine plus azithromycin may improve birth outcomes through impacts on inflammation and placental angiogenesis independent of malarial infection
Source: Sci Rep. 2019 Feb 19;9:2260. doi: 10.1038/s41598-019-38821-2 (PMC6381158; doi:10.1038/s41598-019-38821-2)

Title: Sulphadoxine-pyrimethamine plus azithromycin may improve birth outcomes through impacts on inflammation and placental angiogenesis independent of malarial infection

Authors:
Holger W. UNGER PhD[1]
Annjaleen P. HANSA MBBS[1,2]
Christelle BUFFET MSc[1]
Wina HASANG BSc[1]
Andrew TEO PhD[1]
Louise RANDALL PhD[1]
Maria OME KAIUS MBBS[3,4,5]
Stephan KARL[3,4,5,6]
Ayen A. ANUAN BSc[1]
James G. BEESON PhD[1,2,7]
Ivo MUELLER PhD[4,5,8]
Sarah J STOCK PhD[9]
Stephen J. ROGERSON FRACP[1]

[1]Department of Medicine at the Doherty Institute, University of Melbourne, Parkville, Victoria, Australia
[2]Central Clinical School and Department of Microbiology, Monash University, Victoria, Australia
[3]Papua New Guinea Institute of Medical Research, Goroka, Papua New Guinea
[4]Walter and Eliza Hall Institute, Parkville, Victoria, Australia
[5]Department of Medical Biology, University of Melbourne, Parkville, Victoria, Australia
[6]Australian Institute of Tropical Health and Medicine, James Cook University, Cairns, Australia
[7]Burnet Institute, Melbourne, Victoria, Australia
[8]Institut Pasteur, Paris, France
[9]Tommy's Centre for Maternal and Fetal Health, MRC Centre for Reproductive Health, Queen's Medical Research Institute, Edinburgh, UK

Supplemental File 1: STROBE Statement—checklist of items that should be included in reports of observational studies

	Item No	Recommendation
**Title and abstract**	1	(*a*) Indicate the study's design with a commonly used term in the title or the abstract<br>**Included in title (cohort study first and foremost)**
		(*b*) Provide in the abstract an informative and balanced summary of what was done and what was found<br>**Abstract**
**Introduction**		
Background/rationale	2	Explain the scientific background and rationale for the investigation being reported<br>**Introduction**
Objectives	3	State specific objectives, including any prespecified hypotheses<br>**Introduction, last paragraph**
**Methods**		
Study design	4	Present key elements of study design early in the paper<br>**Methods, first paragraph**
Setting	5	Describe the setting, locations, and relevant dates, including periods of recruitment, exposure, follow-up, and data collection<br>**Methods, second paragraph. Parent trial paper.**
Participants	6	(*a*) *Cohort study*—Give the eligibility criteria, and the sources and methods of selection of participants. Describe methods of follow-up<br>*Case-control study*—Give the eligibility criteria, and the sources and methods of case ascertainment and control selection. Give the rationale for the choice of cases and controls<br>*Cross-sectional study*—Give the eligibility criteria, and the sources and methods of selection of participants<br>**Methods, second paragraph. Parent trial paper.**
		(*b*) *Cohort study*—For matched studies, give matching criteria and number of exposed and unexposed<br>*Case-control study*—For matched studies, give matching criteria and the number of controls per case
Variables	7	Clearly define all outcomes, exposures, predictors, potential confounders, and effect modifiers. Give diagnostic criteria, if applicable<br>**Methods, statistical analyses**
Data sources/ measurement	8*	For each variable of interest, give sources of data and details of methods of assessment (measurement). Describe comparability of assessment methods if there is more than one group<br>**Methods**
Bias	9	Describe any efforts to address potential sources of bias<br>**Methods, statistical analyses**
Study size	10	Explain how the study size was arrived at<br>**Methods, second paragraph**
Quantitative variables	11	Explain how quantitative variables were handled in the analyses. If applicable, describe which groupings were chosen and why<br>**Methods, statistical analyses**
Statistical methods	12	(*a*) Describe all statistical methods, including those used to control for confounding<br>**Methods, statistical analyses**

		(*b*) Describe any methods used to examine subgroups and interactions<br>**Methods, statistical analyses**
		(*c*) Explain how missing data were addressed<br>**Methods, statistical analyses**
		(*d*) *Cohort study*—If applicable, explain how loss to follow-up was addressed<br>**Original trial paper.**<br>*Case-control study*—If applicable, explain how matching of cases and controls was addressed<br>*Cross-sectional study*—If applicable, describe analytical methods taking account of sampling strategy
		(*e*) Describe any sensitivity analyses
**Results**		
Participants	13*	(a) Report numbers of individuals at each stage of study—eg numbers potentially eligible, examined for eligibility, confirmed eligible, included in the study, completing follow-up, and analysed<br>**Results, Figure 1, parent trial paper.**
		(b) Give reasons for non-participation at each stage<br>**Results, Figure 1, parent trial paper.**
		(c) Consider use of a flow diagram<br>**Figure 1**
Descriptive data	14*	(a) Give characteristics of study participants (eg demographic, clinical, social) and information on exposures and potential confounders<br>**Table 1**
		(b) Indicate number of participants with missing data for each variable of interest<br>**Table 1, results.**
		(c) *Cohort study*—Summarise follow-up time (eg, average and total amount)<br>**Until delivery/known pregnancy outcome. Parent trial paper.**
Outcome data	15*	*Cohort study*—Report numbers of outcome events or summary measures over time<br>**Tables 1&2, results.**
		Case-control study—Report numbers in each exposure category, or summary measures of exposure
		Cross-sectional study—Report numbers of outcome events or summary measures
Main results	16	(*a*) Give unadjusted estimates and, if applicable, confounder-adjusted estimates and their precision (eg, 95% confidence interval). Make clear which confounders were adjusted for and why they were included<br>**Methods, statistical analyses. Results, Tables 1-3, Supp Tables 1&2.**
		(*b*) Report category boundaries when continuous variables were categorized<br>**Methods, statistical analyses. Results, Tables 1-3, Supp Tables 1&2.**
		(*c*) If relevant, consider translating estimates of relative risk into absolute risk for a meaningful time period
Other analyses	17	Report other analyses done—eg analyses of subgroups and interactions, and sensitivity analyses<br>**Methods, Results.**
**Discussion**		
Key results	18	Summarise key results with reference to study objectives<br>**Discussion, Principal findings.**
Limitations	19	Discuss limitations of the study, taking into account sources of potential bias or imprecision. Discuss both direction and magnitude of any potential bias<br>**Discussion, Strengths and limitations**
Interpretation	20	Give a cautious overall interpretation of results considering objectives, limitations, multiplicity of analyses, results from similar studies, and other relevant evidence<br>**Discussion, Meaning and implication of study findings.**
Generalisability	21	Discuss the generalisability (external validity) of the study results<br>**Discussion**
**Other information**		

Funding	22	Give the source of funding and the role of the funders for the present study and, if applicable, for the original study on which the present article is based<br>**Funding declaration**

*Give information separately for cases and controls in case-control studies and, if applicable, for exposed and unexposed groups in cohort and cross-sectional studies.

Note: An Explanation and Elaboration article discusses each checklist item and gives methodological background and published examples of transparent reporting. The STROBE checklist is best used in conjunction with this article (freely available on the Web sites of PLoS Medicine at http://www.plosmedicine.org/, Annals of Internal Medicine at http://www.annals.org/, and Epidemiology at http://www.epidem.com/). Information on the STROBE Initiative is available at www.strobe-statement.org.

Supplemental Table 1 Association of maternal clinical factors at enrolment and delivery with AGP (α-1-Acid glycoprotein) and CRP (C-reactive protein) levels at the time of clinical assessment

	CRP (mg/L)		*P*	AGP (mg/L)		*P*
Enrolment	Yes	No		Yes	No	
Feeling unwell	2.7 (1.6, 4.4)	1.4 [1.3, 1.5]	<0.001	265 (213, 329)	220 [212, 228]	NS
History of fever (last 24 hours)	3.2 (1.9, 5.4)	1.4 (1.3, 1.5)	<0.001	252 (206, 310)	220 (212, 228)	NS
Patient-reported malaria	1.9 (1.5, 2.3)	1.4 (1.3, 1.4)	0.001	253 (228, 281)	217 (209, 226)	0.006
Malaria infection[a]	2.5 (2.0, 3.0)	1.3 (1.2, 1.4)	<0.001	241 (217, 267)	218 (210, 227)	NS
Sexually transmitted infection[b]	1.5 (1.1, 2.1)	1.3 (1.1, 1.6)	NS	198 (160, 244)	189 (165, 217)	NS
MUAC <23 cm	1.2 (1.1, 1.4)	1.5 (1.4, 1.6)	0.007	214 (200, 229)	224 (214, 234)	NS
MAP ≥90mmHg	1.3 (12, 1.5)	1.4 (1.3, 1.5)	NS	239 (224, 255)	216 (207, 225)	0.020
Haemoglobin <70 g/L	2.5 (1.7, 3.8)	1.4 (1.3, 1.5)	<0.001	261 (217, 312)	217 (209, 226)	NS
Delivery[c]	Yes	No		Yes	No	
Peripheral malaria infection (any species)[c]	2.5 (1.9, 3.3)	1.1 (1.0, 1.2)	<0.001	187 (160, 219)	167 (162, 173)	NS
Placental malaria[c]	2.3 (1.5, 3.5)	1.0 (0.9, 1.1)	<0.001	165 (133, 204)	162 (156, 168)	NS
Active placental infection (histology)	1.7 (1.2, 2.4)	1.0 (0.9, 1.1)	<0.001	193 (167, 223)	160 (153, 167)	0.012
Past placental infection (histology)	1.3 (1.0, 1.7)	0.9 (0.9, 1.0)	0.020	151 (96, 134)	161 (155, 168)	NS

MAP, mean arterial pressure; *MUAC*, mid-upper arm circumference; *NS*, not significant, defined as p≥0.05. Data are presented as geometric mean (95% confidence interval).

[a] Peripheral parasitaemia by light microscopy and/or real-time polymerase chain reaction (*Plasmodium falciparum*, *P. vivax*).
[b] One or more of the following detected in self-collected low vaginal swab: *Trichomonas vaginalis*, *Chlamydia trachomatis*, *Neisseria gonorrhoeae* (n=309)
[c] Positive for infection by light microscopy and/or polymerase chain reaction (*P. falciparum, P.vivax*)

Supplemental table 2 Association of maternal clinical factors at enrolment and delivery with levels of soluble endoglin (sEng), s-Flt-1 (soluble fms-like tyrosine kinase), PlGF (placental growth factor) and the sFlt-1/PlGF ratio at the time of clinical assessment.

	sEng (pg/ml)			sFlt-1 (ng/ml)		
**Enrolment**	Yes	No	*P*	Yes	No	*P*
Feeling unwell	14.8 (12.5, 17.4)	14.7 (14.2, 15.2)	NS	2.6 (2.1, 3.1)	2.6 (2.5, 2.7)	NS
History of fever (last 24 hours)	14.3 (11.8, 17.3)	14.7 (14.2, 15.2)	NS	2.4 (1.9, 2.9)	2.6 (2.5, 2.7)	NS
Patient-reported malaria	15.3 (13.9, 16.7)	14.6 (14.1, 15.2)	NS	2.6 (2.4, 2.9)	2.5 (2.5, 2.7)	NS
Malaria infection[a]	15.8 (14.4, 17.2)	14.6 (14.0, 15.1)	NS	2.3 (2.1, 2.5)	2.6 (2.5, 2.7)	0.017
MUAC <23 cm	14.6 (13.7, 15.5)	14.8 (14.2, 15.4)	NS	2.8 (2.6, 3.0)	2.5 (2.4, 2.6)	0.018
MAP ≥90mmHg	16.3 (15.2, 17.4)	14.3 (13.7, 14.8)	<0.001	2.5 (2.4, 2.7)	2.6 (2.5, 2.7)	NS
Haemoglobin <70 g/L	15.9 (13.4, 19.0)	14.7 (14.2, 15.2)	NS	3.0 (2.4, 3.8)	2.5 (2.4, 2.6)	NS
**Delivery**[b]	Yes	No	*P*	Yes	No	*P*
Peripheral malaria infection (any species)[b]	21.9 (18.4, 26.1)	22.9 (21.9, 23.9)	NS	4.8 (3.8, 5.9)	4.8 (4.5, 5.1)	NS
Placental malaria[b]	24.7 (19.5, 31.4)	23.5 (22.4, 24.6)	NS	5.6 (4.3, 7.3)	6.3 (5.9, 6.7)	NS
Active placental infection (histology)	23.6 (19.6, 28.5)	23.4 (22.2, 24.7)	NS	6.7 (5.4, 8.4)	6.2 (5.8, 6.6)	NS
Past placental infection (histology)	18.9 (16.1, 31.7)	24.1 (22.8, 25.4)	0.003	4.9 (4.0, 6.0)	6.4 (5.9, 6.8)	0.013

	PlGF (pg/ml)			sFlt-1/PlGF		
**Enrolment**	Yes	No	*P*	Yes	No	*P*
Feeling unwell	123 (103, 155)	161 (155, 167)	0.017	20.4 (15.1, 27.7)	16.0 (15.2, 16.8)	NS
History of fever (last 24 hours)	146 (120, 177)	160 (154, 166)	NS	16.2 (12.2, 21.5)	16.1 (15.3, 16.9)	NS
Patient reported malaria	153 (139, 169)	160 (154, 166)	NS	17.2 (15.1, 19.6)	16.0 (15.1, 16.8)	NS
Malaria infection[a]	141 (128, 154)	163 (156, 169)	0.006	16.3 (14.4, 18.5)	16.1 (15.2, 16.9)	NS
MUAC <23 cm	157 (146, 168)	160 (154, 167)	NS	17.5 (16.0, 19.3)	15.6 (14.7, 16.5)	0.031
MAP ≥90mmHg	157 (146, 169)	160 (154, 167)	NS	16.2 (14.6, 17.8)	16.1 (15.2, 17.0)	NS
Haemoglobin <70 g/L	137 (115, 163)	160 (154, 166)	NS	22.1 (17.0, 28.8)	15.9 (15.0, 16.7)	0.011
	Yes	No	*P*	Yes	No	*P*
**Delivery**[b]						
Peripheral malaria infection (any species)[c]	74 (65, 84)	76 (73, 78)	NS	64.4 (50.6, 82.0)	63.6 (59.7, 67.9)	NS

Placental malaria[b]	80 (67, 96)	80 (77, 82)	NS	70.5 (50.4, 98.7)	78.6 (73.7, 84.0)	NS
Active placental infection (histology)	81 (71, 93)	77 (74, 80)	NS	83.1 (65.5, 105.4)	80.3 (74.7, 86.3)	NS
Past placental infection (histology)	79 (71, 89)	77 (74, 80)	NS	61.6 (49.4, 76.9)	83.2 (77.1, 89.8)	0.008

MAP, mean arterial pressure; *MUAC*, mid-upper arm circumference; *NS*, not significant, defined as p≥0.05. Data are presented as geometric mean (95% confidence interval).
[a] Peripheral parasitaemia by light microscopy and/or real-time polymerase chain reaction (*Plasmodium falciparum*, *P. vivax*).
[b] Positive for infection by light microscopy and/or polymerase chain reaction (*P. falciparum, P.vivax*)

Supplemental table 3 Biomarker levels at enrolment and birthweight, overall and by treatment arm

Biomarker levels		N	Mean birthweight in g (95% CI)	Adjusted mean difference in BW (g) (95% CI)	*P*
**Overall**					
CRP (mg/L)		1,647	2951	51 (-2, 104)	NS
	Elevated (≥5.0)	365	2907		
AGP (mg/L)		1,509	2951	34 (-14, 81)	NS
	Elevated (≥360)	503	2918		
sEng (pg/ml)		1,509	2956	38 (-9, 85)	NS
	Elevated (≥28.3)	503	2903		
sFlt-1 (ng/ml)		1,509	2955	23 (-25, 70)	NS
	Elevated (≥4.3)	503	2909		
PlGF (pg/ml)		1,509	2957	47 (-2, 47)	NS
	Low (≤93.5)	503	2901		
sFlt-1/PlGF		1,509	2960	45 (-3, 93)	NS
	Elevated (≥38)	503	2894		
**SPCQ**					
CRP (mg/L)		837	2937	96 (13, 179)	0.023
	Elevated (≥5.0)	166	2842		
AGP (mg/L)		740	2927	21 (-50, 92)	NS
	Elevated (≥360)	240	2906		
sEng (pg/ml)		756	2936	37 (-35, 109)	NS
	Elevated (≥28.3)	247	2880		
sFlt-1 (ng/ml)		742	2944	65 (-6, 135)	NS
	Elevated (≥4.3)	261	2860		
PlGF (pg/ml)		748	2931	38 (-35, 112)	NS
	Low (≤93.5)	255	2897		
sFlt-1/PlGF		761	2941	61 (-13, 134)	NS
	Elevated (≥38)	242	2865		
**SPAZ**					
CRP (mg/L)		810	2965	15 (-53, 83)	NS
	Elevated (≥5.0)	199	2961		
AGP (mg/L)		769	2974	43 (-21, 106)	NS
	Elevated (≥360)	240	2964		
sEng (pg/ml)		753	2977	40 (-22, 103)	NS
	Elevated (≥28.3)	256	2928		
sFlt-1 (ng/ml)		767	2965	18 (-45, 82)	NS

	Elevated (≥4.3)	242	2963		
PlGF (pg/ml)		761	2983	58 (-7, 123)	NS
	Low (≤93.5)	248	2905		
sFlt-1/PlGF		748	2979	35 (-28, 97)	NS
	Elevated (≥38)	261	2921		

AGP, α-1-Acid glycoprotein; *aOR*, adjusted odds ratio; *CI*, confidence interval; *CRP*, C-reactive protein; *PlGF*, placental growth factor; *sEng*; soluble endoglin; *sFlt-1*, soluble fms-like tyrosine kinase; *SPCQ*, sulfadoxine-pyrimethamine plus chloroquine; *SPAZ,* sulfadoxine-pyrimethamine plus azithromycin. *NS*, not significant, defined as $p \geq 0.05$.

[a] Odds ratios and 95% confidence intervals were estimated using logistic regression and adjusted for sex, bed net use, mid-upper arm circumference, recruitment clinic, height, partner's work status, number of antenatal visits, timing of birthweight measurement, and fundal height at biomarker measurement

[b] Odds ratios and 95% confidence intervals were estimated using logistic regression and adjusted for sex, bed net use, mid-upper arm circumference, recruitment clinic, height, partner's work status, number of antenatal visits, and gestational age at biomarker measurement by ultrasound

Supplemental Table 4 Adjusted odds ratios (aOR) for biomarker levels at enrolment (log-transformed) and adverse birth outcomes, overall and by trial arm, in women without detectable *Plasmodium falciparum*/*P. vivax* infection.

**Outcome**	**SPCQ**		**SPAZ**		**All**	
	aOR (95% CI)	*P*	aOR (95% CI)	*P*	aOR (95% CI)	*P*
**Low birthweight**[a]	**128/744**		**103/796**		**231/1,540**	
CRP (mg/L)	1.10 (0.80, 1.51)	0.56	1.00 (0.70, 1.44)	1.00	1.04 (0.82, 1.32)	0.73
AGP (mg/L)	0.86 (0.50, 1.50)	0.60	1.66 (0.87, 3.16)	0.13	1.15 (0.76, 1.74)	0.51
sEng (pg/ml)	1.47 (0.78, 2.78)	0.23	1.38 (0.72, 2.67)	0.33	1.45 (0.93, 2.28)	0.10
sFlt-1 (ng/ml)	1.39 (0.83, 2.35)	0.21	1.86 (0.97, 3.57)	0.06	1.53 (1.02, 2.29)	0.041
PlGF (pg/ml)	0.64 (0.35, 1.16)	0.14	0.78 (0.41, 1.48)	0.44	0.70 (0.45, 1.08)	0.11
sFlt-1/PlGF	1.56 (1.02, 2.37)	0.039	1.58 (0.99, 2.54)	0.06	1.54 (1.13, 2.10)	0.006
**Preterm birth**[b]	**51/484**		**34/521**		**85/1,005**	
CRP (mg/L)	2.30 (1.39, 3.79)	0.001	0.80 (0.43, 1.47)	0.46	1.45 (0.99, 2.12)	0.06
AGP (mg/L)	1.17 (0.49, 2.78)	0.72	0.90 (0.33, 2.44)	0.84	1.03 (0.55, 1.96)	0.92
sEng (pg/ml)	3.77 (1.32, 10.7)	0.013	0.61 (0.19, 1.94)	0.40	1.70 (0.82, 3.56)	0.16
sFlt-1 (ng/ml)	2.50 (1.10, 5.60)	0.029	1.68 (0.63, 4.50)	0.31	2.08 (0.11, 3.89)	0.02
PlGF (pg/ml)	1.92 (0.73, 5.08)	0.19	0.55 (0.17, 1.75)	0.31	1.14 (0.55, 2.37)	0.73
sFlt-1/PlGF	1.33 (0.70, 2.51)	0.38	1.72 (0.82, 3.60)	0.15	1.46 (0.90, 2.35)	0.13
**SGA**[b]	**120/484**		**120/521**		**240/1,005**	
CRP (mg/L)	1.09 (0.77, 1.53)	0.64	0.97 (0.68, 1.39)	0.88	1.02 (0.80, 1.30)	0.89
AGP (mg/L)	0.98 (0.56, 1.74)	0.95	1.42 (0.78, 2.57)	0.25	1.18 (0.79, 1.78)	0.42
sEng (pg/ml)	0.75 (0.39, 1.45)	0.39	1.02 (0.52, 2.00)	0.95	0.89 (0.56, 1.42)	0.63
sFlt-1 (ng/ml)	0.89 (0.50, 1.60)	0.70	1.15 (0.63, 2.11)	0.65	1.01 (0.65, 1.52)	0.97
PlGF (pg/ml)	0.53 (0.25, 1.11)	0.09	0.55 (0.27, 1.11)	0.10	0.56 (0.34, 0.94)	0.026
sFlt-1/PlGF	1.20 (0.76, 1.92)	0.44	1.42 (0.89, 2.25)	0.14	1.28 (0.92, 1.77)	0.14

AGP, α-1-acid glycoprotein; *CI*, confidence interval; *CRP*, C-reactive protein; *PlGF*, placental growth factor; *sEng*; soluble endoglin; *sFlt-1*, soluble fms-like tyrosine kinase; *SPCQ*, sulphadoxine-pyrimethamine plus chloroquine; *SPAZ,* sulphadoxine-pyrimethamine plus azithromycin. *NS*, not significant, defined as p≥0.05.

[a] Odds ratios and 95% confidence intervals were estimated using logistic regression and adjusted for sex, bed net use, mid-upper arm circumference, recruitment clinic, height, partner's work status, number of antenatal visits, timing of birthweight measurement, and fundal height at biomarker measurement
[b] Odds ratios and 95% confidence intervals were estimated using logistic regression and adjusted for sex, bed net use, mid-upper arm circumference, recruitment clinic, height, partner's work status, number of antenatal visits, and gestational age at biomarker measurement by ultrasound

Supplemental Table 5 Adjusted odds ratios (aOR) for biomarker levels at delivery (log-transformed) and adverse birth outcomes, overall and by trial arm, in women without detectable *Plasmodium falciparum*/*P. vivax* infection.

**Outcome**	**SPCQ**		**SPAZ**		**All**	
	aOR (95% CI)	*P*	aOR (95% CI)	*P*	aOR (95% CI)	*P*
**Low birthweight**[a]	**122/712**		**99/769**		**221/1,481**	
CRP (mg/L)	1.21 (0.90, 1.66)	0.21	0.90 (0.64, 1.27)	0.56	1.09 (0.87, 1.36)	0.47
AGP (mg/L)	2.47 (1.29, 4.73)	0.007	1.92 (0.97, 3.80)	0.06	2.16 (1.36, 3.45)	0.001
sEng (pg/ml)	2.34 (1.31, 4.20)	0.004	2.03 (1.07, 3.84)	0.030	2.15 (1.40, 3.29)	0.001
sFlt-1 (ng/ml)	1.29 (0.66, 2.54)	0.46	0.95 (0.44, 2.04)	0.90	1.24 (0.76, 2.01)	0.39
PlGF (pg/ml)	0.50 (0.24, 1.05)	0.07	0.57 (0.25, 1.27)	0.17	0.53 (0.31, 0.91)	0.021
sFlt-1/PlGF	1.16 (0.81, 1.65)	0.43	1.70 (1.10, 2.63)	0.017	1.34 (1.02, 1.76)	0.036
**Preterm birth**[b]	**50/464**		**33/505**		**83/969**	
CRP (mg/L)	1.42 (0.87, 2.32)	0.16	0.52 (0.28, 0.95)	0.035	0.99 (0.69, 1.42)	0.95
AGP (mg/L)	4.27 (1.53, 11.7)	0.005	1.35 (0.45, 4.01)	0.59	2.47 (1.20, 5.07)	0.014
sEng (pg/ml)	3.02 (1.26, 7.22)	0.013	3.90 (1.24, 12.3)	0.020	3.06 (1.55, 6.07)	0.001
sFlt-1 (ng/ml)	0.99 (0.57, 1.71)	0.97	1.82 (0.83, 3.97)	0.13	1.23 (0.79, 1.90)	0.37
PlGF (pg/ml)	0.29 (0.09, 0.97)	0.045	0.49 (0.12, 1.98)	0.32	0.34 (0.14, 0.86)	0.02
sFlt-1/PlGF	1.30 (0.77, 2.20)	0.33	1.92 (0.96, 3.86)	0.07	1.51 (1.00, 2.28)	0.05
**SGA**[b]	**117/464**		**117/505**		**234/969**	
CRP (mg/L)	1.31 (0.94, 1.82)	0.11	0.97 (0.70, 1.35)	0.86	1.12 (0.89, 1.41)	0.35
AGP (mg/L)	3.94 (1.91, 8.14)	<0.001	1.41 (0.75, 2.66)	0.29	2.22 (1.39, 3.56)	0.001
sEng (pg/ml)	1.82 (1.04, 3.18)	0.035	1.19 (0.70, 2.02)	0.53	1.44 (0.98, 2.10)	0.06
sFlt-1 (ng/ml)	1.24 (0.84, 1.82)	0.29	1.19 (0.78, 1.82)	0.42	1.20 (0.91, 1.60)	0.20
PlGF (pg/ml)	0.98 (0.48, 2.02)	0.96	0.82 (0.38, 1.77)	0.62	0.93 (0.55, 1.56)	0.78
sFlt-1/PlGF	1.21 (0.84, 1.74)	0.31	1.23 (0.83, 1.81)	0.31	1.20 (0.92, 1.56)	0.18

AGP, α-1-Acid glycoprotein; *CI*, confidence interval; *CRP*, C-reactive protein; *PlGF*, placental growth factor; *sEng*; soluble endoglin; *sFlt-1*, soluble fms-like tyrosine kinase; *SPCQ*, sulphadoxine-pyrimethamine plus chloroquine; *SPAZ,* sulphadoxine-pyrimethamine plus azithromycin. *NS*, not significant, defined as p≥0.05.

[a] Odds ratios and 95% confidence intervals were estimated using logistic regression and adjusted for sex, bed net use, mid-upper arm circumference, recruitment clinic, height, partner's work status, number of antenatal visits, and timing of birthweight measurement
[b] Odds ratios and 95% confidence intervals were estimated using logistic regression and adjusted for sex, bed net use, mid-upper arm circumference, recruitment clinic, height, partner's work status, and number of antenatal visits

Supplemental Table 6 Diagnostic characteristics of biomarkers for the detection of low birthweight (<2,500 g), spontaneous preterm birth (PTB, <37 weeks) and birthweight <10 birthweight centile (SGA) among women randomised to SPCQ

**LBW**	**Prevalence:**	17.5% (15.0, 19.9)		175/1003					
	AUC	Youden Index (YI)	Biomarker level at YI	Sensitivity (95% CI)	Specificity (95% CI)	Positive predictive value (95% CI)	Negative predictive value (95% CI)	+LR	-LR
**LBW**									
CRP	0.5186	0.08	6.1 mg/L	18.3 (12.9, 24.8)	88.2 (85.8, 90.3)	24.6 (17.5, 32.9)	83.6 (81.0, 86.0)	1.54	0.93
AGP	0.5067	0.06	265 mg/L	37.7 (30.5, 45.3)	56.3 (52.8, 59.7)	15.4 (12.1, 19.2)	81.0 (77.6, 84.2)	0.86	1.11
sEng	0.5693	0.13	21.5 pg/mL	49.7 (42.1, 57.4)	62.3 (58.9, 65.6)	21.8 (17.8, 26.2)	85.4 (82.4, 88.1)	1.32	0.81
sFlt-1	0.5356	0.10	4.2 ng/mL	35.4 (28.4, 43.0)	74.6 (71.5, 77.6)	22.8 (17.9, 28.2)	84.5 (81.7, 87.1)	1.40	0.87
PlGF	0.5264	0.07	336 pg/ml	13.7 (9.0, 19.7)	80.1 (77.2, 82.7)	12.7 (8.3, 18.3)	81.4 (78.6, 84.1)	1.08	0.64
sFlt1/PGF	0.5472	0.10	29.9	35.4 (28.4, 43.0)	74.0 (70.9, 77.0)	22.4 (17.6, 27.8)	84.4 (81.6, 87.0)	1.36	0.87
**PTB**		10.6% (8.4, 13.3)		69/649					
CRP	0.5678	0.14	6.6 mg/L	21.7 (12.7, 33.3)	90.7 (88.0, 92.8)	21.7 (12.7, 33.3)	90.7 (88.0, 92.9)	2.33	0.86
AGP	0.5327	0.13	244 mg/L	58.0 (45.5, 69.8)	53.3 (49.1, 57.4)	12.9 (9.4, 17.1)	91.4 (87.9, 94.2)	1.24	0.79
sEng	**0.6310**	0.22	25.3 pg/mL	49.3 (37.0, 61.6)	71.4 (67.5, 75.0)	17.0 (12.1, 22.9)	92.2 (89.3, 94.5)	1.72	0.71
sFlt-1	0.6072	0.21	2.7 ng/mL	65.2 (52.8, 76.3)	55.3 (51.2, 59.4)	14.8 (11.0, 19.3)	93.0 (89.8, 95.5)	1.46	0.63
PlGF	0.5240	0.11	60.6 pg/ml	95.7 (87.8, 99.1)	13.4 (10.8, 16.5)	11.6 (9.1, 14.5)	96.3 (89.6, 99.2)	1.11	0.32
sFlt1/PGF	0.5539	0.12	14.4	63.8 (51.3, 75.0)	47.1 (42.9, 51.2)	12.5 (9.3, 1.5)	91.6 (87.9, 84.5)	1.20	0.77
**SGA**		25.1% (22.0, 28.6)		163/649					
CRP	0.4960	0.05	1.3 mg/L	47.9 (40.0, 55.8)	56.0 (51.4, 60.4)	26.7 (21.7, 32.2)	76.2 (71.4, 80.5)	1.09	0.93
AGP	0.5135	0.05	406 mg/L	23.9 (17.6, 31.2)	80.7 (76.9, 84.1)	29.3 (21.8, 37.8)	76.0 (72.0, 79.6)	1.24	0.94
sEng	0.5078	0.06	11.1 pg/mL	65.0 (57.2, 72.3)	40.9 (36.5, 45.5)	27.0 (22.6, 31.6)	77.7 (72.1, 82.7)	1.1	0.85
sFlt-1	0.5120	0.07	1.2 ng/mL	79.8 (72.8, 85.6)	13.6 (10.7, 17.0)	23.6 (20.1, 27.4)	66.7 (56.5, 75.8)	0.92	1.49
PlGF	0.5624	0.13	80.3 pg/ml	74.2 (66.8, 80.8)	13.6 (10.7, 17.0)	22.4 (18.9, 26.1)	61.1 (51.3, 70.3)	0.86	1.90
sFlt1/PGF	0.5382	0.09	38.9	26.4 (19.8, 33.8)	82.1 (78.4, 85.4)	33.1 (25.1, 41.9)	76.9 (73.0, 80.4)	1.47	0.90

AGP, α-1-Acid glycoprotein; *AUC*, area under the curve (receiver-operator curve); *CI*, confidence interval; *CRP*, C-reactive protein; *LR*, likelihood ratio; *PlGF*, placental growth factor; *sEng*; soluble endoglin; *sFlt-1*, soluble fms-like tyrosine kinase; SPCQ; sulfadoxine-pyrimethamine plus chloroquine.

The best combination for prediction of PTB is sEng, sFLt-1 and maternal mid-upper arm circumference, which provides an AUC of 0.686.

Supplemental Figure 1. Comparison of levels of α-1-acid glycoprotein (AGP) between treatment arms by gestational age (GA) groupings.

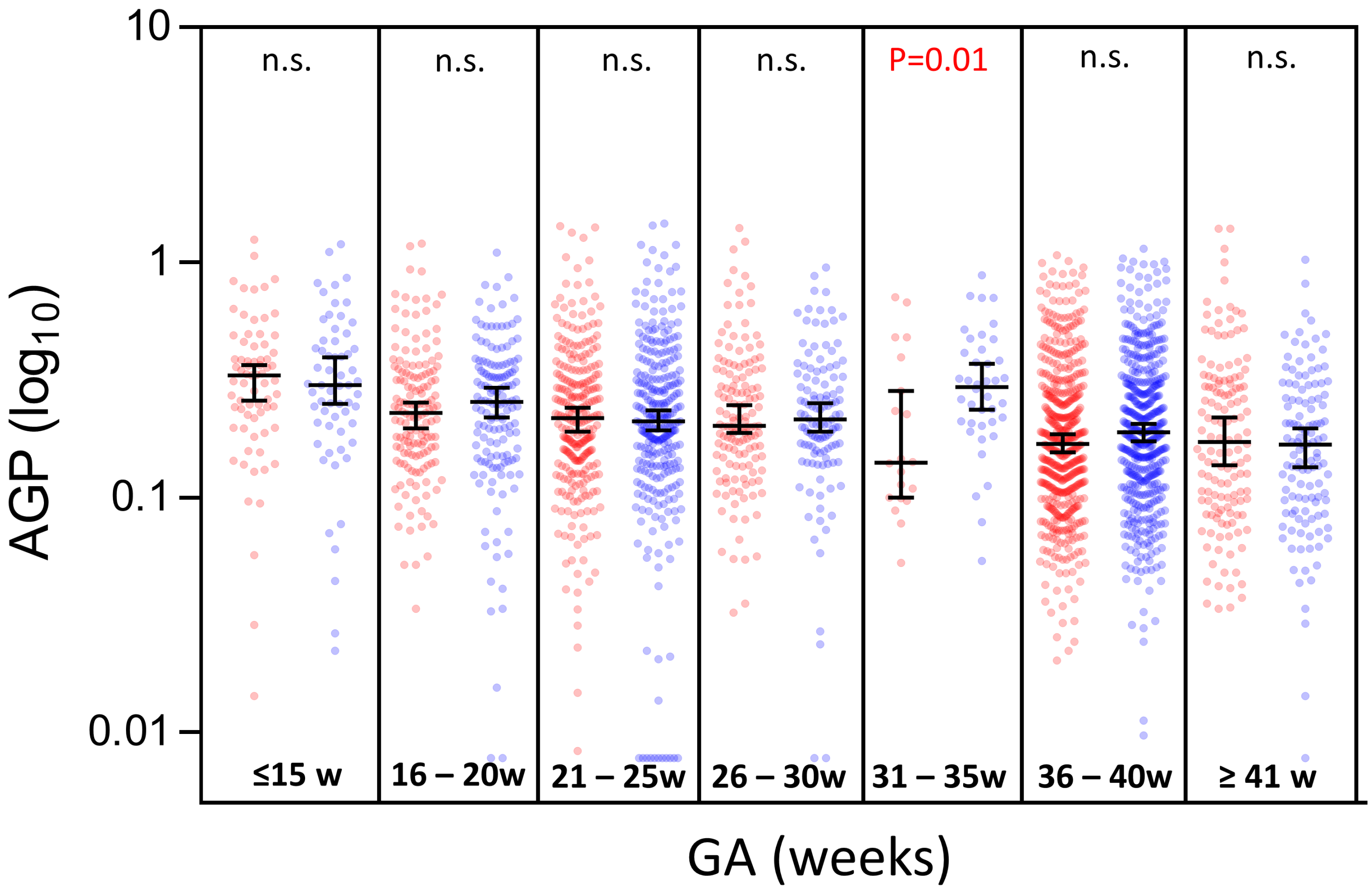

Supplement: Supplementary file 1 — Supplemental Information [file 41598_2019_38821_MOESM1_ESM.pdf]
